# Application of WeChat Platform in Midterm Clinical Follow-Up of Children Who Underwent Transthoracic Device Closure of VSD

**DOI:** 10.21470/1678-9741-2020-0376

**Published:** 2022

**Authors:** Wen-Peng Xie, Jian-Feng Liu, Yu-Qing Lei, Zeng-Chun Wang, Qiang Chen, Hua Cao

**Affiliations:** 1 Department of Cardiac Surgery, Fujian Maternity and Child Health Hospital, Affiliated Hospital of Fujian Medical University, Fuzhou, People’s Republic of China.; 2 Fujian Key Laboratory of Women and Children’s Critical Diseases Research, Fujian Maternity and Child Health Hospital, Fuzhou, People’s Republic of China.; 3 Department of Cardiovascular Surgery, Union Hospital, Fujian Medical University, Fuzhou, People’s Republic of China.

**Keywords:** Follow-Up, VSD, Device, Social Media

## Abstract

**Introduction:**

The purpose of this study was to investigate the feasibility and superiority of using the WeChat platform for midterm clinical follow-up of children who underwent transthoracic device closure for ventricular septal defects (VSDs).

**Methods:**

Ninety children with VSDs who underwent transthoracic device closure were divided into a WeChat follow-up group (WFU group) and an outpatient follow-up group (OFU group). The patients were followed up via WeChat or at an outpatient clinic three months and one year after discharge. The incidences of adverse events, associated complications, costs and time spent, loss to follow-up rate, medication adherence, and overall satisfaction were recorded.

**Results:**

There was no statistically significant difference in the incidence of adverse events or postoperative complications between the two groups. Also, the loss to follow-up rate was similar between them. Compared with the OFU group, there were significant statistical advantages in the WFU group regarding the total time and cost spent, medication adherence, and satisfaction.

**Conclusion:**

The use of the WeChat platform in midterm clinical follow-up of children who underwent transthoracic device closure for VSDs has the advantages of reducing financial and time burdens, facilitating high medication adherence, and leading to high satisfaction.

**Table:** 

Abbreviations, acronyms & symbols	
**CNY**	**= China Yuan**
**OFU**	**= Outpatient follow-up**
**VSD**	**= Ventricular septal defects**
**WFU**	**= WeChat follow-up**

## INTRODUCTION

Ventricular septal defects (VSDs) are some of the most common congenital heart diseases, accounting for 40% of these^[1]^. The traditional treatment for VSDs is surgical repair via cardiopulmonary bypass, which has the disadvantages of large surgical trauma, a long recovery time, and scarring^[2]^. Another common treatment is transcatheter device closure of the VSD, which has the advantages of no incision and a fast recovery; however, radiation exposure is required^[3]^. In recent years, transthoracic device closure has been widely used to treat VSDs in China. This technique combines the advantages of the two treatments abovementioned, such as a fast recovery, no radiation exposure, and small wounds^[4]^, but the following complications still exist: 1. late-onset complete atrioventricular block; 2. occluder dislodgement; 3. new valve damage; and 4. other late-onset complications^[5-7]^. Therefore, these patients should be followed up closely, and aspirin should be taken orally for approximately three months as an anticoagulant. With the increased detection rate and operation volume among children with congenital heart disease in China, the relative lack of medical resources and the inconvenience of transportation will affect the treatment and follow-up of those children, making it difficult to detect and treat related complications in time^[8]^. Recently, the development of information technology has led to a variety of new medical consultation methods^[9]^. In China, more than 95% of adults own mobile smartphones, and more than 1 billion people use WeChat at least once a day; thus, this platform is deeply integrated into the daily lives of Chinese people^[10]^. Due to the widespread use of the WeChat platform in China, it has emerged as a health education tool for disease management, such as cancer, chronic diseases, and infectious diseases^[10-12]^. Therefore, in this study, we explored the feasibility and superiority of using the WeChat platform in midterm clinical follow-up.

## METHODS

### Research Participants

According to the results of a presurvey, the alpha value was set to 0.05, and the power was set to 0.90. Assuming a certain loss to follow-up rate, we determined that the minimum sample size was 90 patients.

From January 2018 to December 2019, 90 children diagnosed with a simple VSD who successfully underwent transthoracic device closure were selected as the research participants. The inclusion criteria for the study participants were as follows: age > 1 year old, who met the indications for transthoracic device closure, successfully underwent transthoracic device closure of a VSD, with good postoperative recovery, and no serious perioperative complications. The parents also had to have mobile smartphones and had to be familiar with the WeChat platform. The family members of patients who were unwilling to complete this study were excluded. Patients and family members with psychiatric or other medical or mental illnesses that might be likely to affect their ability to agree and participate in the study were also excluded. The study was approved by the ethics committee, and all eligible families provided informed consent. Basic information about the children is shown in Table 1.

**Table 1:** Selected baseline characteristics of the patients.

Characteristics	WFU (n=45)	OFU (n=45)	*P*-value
Age (years)	3.27±1.22	3.21±1.21	0.829
Male (%)	27 (60.00)	29 (64.44)	0.828
VSD size (mm)	4.55±1.89	4.58±1.57	0.696
VSD occluder size (mm)	5.00±2.30	5.68±1.71	0.726
City	16 (35.56)	19 (42.22)	0.666
Rural	29 (64.44)	26 (57.78)	
Parents' education (%)	­		
Less than high school	25 (55.56)	27 (60.00)	0.831
High school and above	20 (44.44)	18 (40.00)	

OFU=outpatient follow-up; VSD=ventricular septal defects; WFU=WeChat follow-up

### Groups

According to the predesigned random distribution envelope, the eligible patients who were discharged from the hospital were randomly divided into the WeChat follow-up group (WFU group, n=45) and the outpatient follow-up group (OFU group, n=45) for clinical follow-up. In each envelope, there was a card with 0 or 1 written on it: 0 represented the WFU group, and 1 represented the OFU group. A laboratory technician who did not directly participate in the study performed a single-blind treatment, so the researchers did not know how the children were randomly grouped.

All patients and their families were given routine discharge and rehabilitation guidance when they were discharged. In the OFU group, routine echocardiography and electrocardiogram examination were performed in the outpatient clinic at three months and at one year of follow-up. The outpatient doctor provided guidance on drugs, diet, and other related matters needing attention. If the patients felt unwell, they could return to the hospital at any time. In the WFU group, routine echocardiography and electrocardiogram examination were performed in the local hospitals at three months and at one year of follow-up, and the related results were uploaded through the WeChat platform by a doctor with a specific WeChat public account dedicated to follow-up work. All family members of children in the WFU group were required to add this specific WeChat account by scanning the QR code to follow the WeChat public account and join the WeChat communication group, and they were taught how to receive and send information. The public account could be used by family members to receive related medical results and to communicate with doctors. Furthermore, this account could be used to send medical information on time every day to remind parents to administer medication to children, and it could be used to regularly send relevant knowledge about postoperative recovery and family health, mainly in the form of texts, pictures, videos, etc. For patients in the WFU group, if they experienced discomfort or abnormal test results, they could be recalled to the hospital immediately for further medical treatment and counseling.

### Postoperative Complications

Postoperative complications were defined as late-onset complete atrioventricular block and malignant arrhythmia, occluder dislodgement, cardiac arrest, etc. Such results in the WFU group were uploaded to the public account, and results in the OFU group were communicated by the outpatient doctor. The evaluation of the echocardiography and electrocardiogram was conducted by a single doctor who was blind to the treatment group. Once occluder dislodgement or malignant arrhythmia occurred, the patients were recalled to the hospital for further examination and treatment.

### Total Time and Cost

In general, the time and cost of follow-up could be measured among both researchers and participants. In our research, we only calculated the time and cost spent by the participants. The time spent by the OFU group was calculated as the time between leaving home and returning home during the follow-up period, and the costs included transportation, accommodation, and inspection costs. For the WFU group, the time spent included not only the inspection time but also the time required to send information and consult with the doctor on the WeChat platform. The financial costs included transportation and inspection fees as well as Internet fees, which are calculated according to China Mobile traffic charges^[13]^. Only the total cost for the WFU group could be calculated because it was difficult to calculate the exact mobile traffic charges for communicating with the doctor.

### Medication Adherence

Anticoagulants needed to be taken for three months, so the medication adherence assessment was performed during the three-month follow-up. Medication adherence was assessed using the four-item Morisky Medication Adherence Scale, which has been used in the study of hypertension, diabetes, and cardiovascular diseases^[14-16]^. The four questions were as follows: 1) Did you ever forget to take your medicine? 2) Were you careless at times about taking your medicine? 3) When you felt better, did you stop taking your medicine? 4) Sometimes if you felt worse when you took the medicine, did you stop taking it? Response options to these four questions were either yes or no and were assigned the values of 1 or 0, respectively. The values were summed to create an adherence score, and a score ≥ 2 was defined as having low medication adherence.

### Loss to Follow-Up Rate

Loss to follow-up is generally considered to be associated with poor outcomes and should be seriously considered when developing a follow-up regimen^[17]^. In this study, if the patients in the OFU group did not respond to the follow-up assessments, they would receive a reminder call. If they were not followed up with after one week, they were deemed to be lost to follow-up. For the WFU group, patients who did not reply in WeChat within three days were given a WeChat message reminder. If they still did not reply within one week, they were considered lost to follow-up. If both the three-month and one-year follow-ups were not completed, the patient was considered lost to follow-up.

### Satisfaction

At the end of the study, the family members were asked to complete the Patient Satisfaction Questionnaire-18 (or PSQ-18) to assess satisfaction in both groups^[18]^.

### Statistical Analysis

All statistical analyses were conducted with IBM Corp. Released 2015, IBM SPSS Statistics for Windows, Version 23.0, Armonk, NY: IBM Corp. Continuous data are expressed as the mean ± standard deviation. The normality of the distributions was assessed for all continuous data, and the data were confirmed to have normal distributions. Clinical parameters between the two groups were compared with the independent samples t-test. The χ^2^ or Fisher’s exact test was used to compare categorical variables. A *P*-value of < 0.05 was defined as statistically significant.

## RESULTS

A total of 86 patients (43 in the WFU group and 43 in the OFU group) completed the study. Two patients in the WFU group withdrew from the study: one patient withdrew due to being unwilling continue the WeChat follow-up at five months and the other patient withdrew due to changes in the WeChat account at seven months. Two patients in the OFU group withdrew from the study: one patient was lost to follow-up at nine months and the other patient withdrew for unknown reasons at six months. The demographic and clinical characteristics of the two groups were not significantly different (Table 1).

Arrhythmia occurred in two patients in the WFU group, including one patient with an incomplete right bundle branch block and one patient with sinus arrhythmia. Similarly, arrhythmia occurred in two patients in the OFU group, including one patient with an incomplete right bundle branch block and one patient with occasional atrial premature beats. There were no occluder dislodgements or other serious complications in both groups.

There were statistically significant differences in the time spent between the WFU group and the OFU group at three months and one year of follow-up (129.59±13.74 *vs*. 394.11±40.23 min, *P*=0.009, and 118.94±17.61 *vs*. 381.63±45.31 min, *P*=0.007, respectively). There were also statistically significant differences in the cost spent between the two groups at three months and one year of follow-up (375.27±42.49 *vs*. 470.07±78.67 China Yuan [CNY], *P*=0.035, and 373.74±41.22 *vs*. 479.35±89.26 CNY, *P*=0.032, respectively) (Tables 2 and 3).

**Table 2:** Comparison of the results between the two groups at the three-month follow-up.

	WFU group	OFU group	*P*-value
Medication adherence	43 (95.56%)	35 (77.78%)	0.027
Time consumption (min)	129.59±13.74	394.11±40.23	0.009
Economic cost (CNY)	375.27±42.49	470.07±78.67	0.035
Lost‑to‑follow‑up rate	0	0	
Satisfaction score	4.28±0.85	3.66±1.02	0.041

CNY=China Yuan; OFU=outpatient follow-up; WFU=WeChat follow-up

**Table 3:** Comparison of the results between the two groups at the one-year follow-up.

	WFU group	OFU group	*P*-value
Medication adherence	-	-	-
Time consumption (min)	118.94±17.61	381.63±45.31	0.007
Economic cost (CNY)	373.74±41.22	479.35±89.26	0.032
Lost‑to‑follow‑up rate	2 (4.44%)	2(4.44%)	1.000
Satisfaction score	4.36±0.76	3.51±1.10	0.037

CNY=China Yuan; OFU=outpatient follow-up; WFU=WeChat follow-up

There was no significant difference in the loss to follow-up rate between the two groups. At the three-month follow-up, two patients in the WFU group had low medication adherence (2/45, 4.44%), and 10 patients in the OFU group had low medication adherence (10/45, 22.2%). Twelve patients in both groups reported that they forgot to take the drug for various reasons. The medication adherence in the WFU group was significantly higher than that in the OFU group (*P*=0.027). Satisfaction in the WFU group was also significantly higher than that in the OFU group at three months and one year of follow-up (4.28±0.85 *vs*. 3.66±1.02, *P*=0.041, and 4.36±0.76 *vs*. 3.51±1.10, *P*=0.037, respectively) (Tables 2 and 3).

## DISCUSSION

VSD is one of the most common congenital heart diseases, and there are many infants and young children with VSD who need treatment every year^[1-4]^. For many Chinese patients with restrictive VSD, transthoracic device closure may be an option. Due to the increasing number of patients with complications after such procedures, increasing attention should be paid to these immediate and long-term complications, especially the occurrence of atrioventricular block^[5-7]^. Therefore, close follow-up is needed to diagnose the complications in time and guarantee the postoperative rehabilitation of the patients. In addition, due to the uneven distribution of medical resources in our country, advanced medical care is mainly concentrated in large cities, and basic medical care in rural areas is relatively lagging. The inconvenience of transportation hinders timely postoperative follow-ups, which might result in an increase in the loss to follow-up rate and make it difficult to detect and treat related complications in time^[8]^. In this study, 61.1% of patients lived in rural areas, and their parents needed to go to hospitals in large cities to seek treatment for their children’s health problems, which greatly increased the financial and time burdens. This usually led the family members of patients to be dissatisfied and worsened the tense doctor-patient relationship. In recent years, mobile medical technology has been widely used as an educational tool for medical guidance^[10,19]^. The WeChat platform can be used as a health education tool for disease management and provides health support by overcoming time and location constraints^[20,21]^. This is a prospective randomized controlled study exploring the feasibility and superiority of using the WeChat platform in the midterm clinical follow-up of patients who underwent transthoracic device closure of a VSD.

With the development of the social economy, Chinese people are paying increasing attention to the time they spend on activities. The results showed that using WeChat for follow-up could greatly save time, which was preferable among patients’ families with a fast pace of life and little free time. In addition, we found that at the three-month follow-up, patients reported spending more time than at the one-year follow-up, which meant that the parents were more familiar in the process of seeing a doctor at the second follow-up. The cost spent in the WFU group was significantly lower than that in the OFU group, which was consistent with the parents’ economic expectations and Chinese medical strategy. Although there was no significant difference in the loss to follow-up rate between these two groups, we found that the family members in the WeChat group were more willing to maintain close contact with the doctor. Through the WeChat platform, it was easier for the parents to obtain professional support. The medication adherence in the WFU group was significantly higher than that in the OFU group, which facilitated the maintenance of effective blood concentration of the anticoagulant drug in the body and reduced the risk of embolism, thereby improving the therapeutic effect and reducing related complications. In addition, the routine reminder from the WeChat platform could effectively reduce the rate of forgetting to take medication. This study showed that the satisfaction of the WFU group was higher than that of the OFU group, possibly because the family members could keep in close contact with the doctor and conveniently communicate with the doctor during the follow-up period. In addition, doctors could give more humane care through the WeChat platform, so the parents might have had a better medical experience, thereby improving the doctor-patient relationship.

It could be inferred that the use of WeChat for patients’ follow-up might have a profound and positive impact on the doctor-patient relationship. Due to big data, the doctor-patient relationship has undergone subtle changes, such that patients have shifted from passive recipients to those who actively obtain health information from the internet, and doctors are also be required to provide more health information^[22,23]^. With the increasing popularity of the internet, network-based health education is considered a viable way to improve the relationship between doctors and patients^[24]^. In this study, via midterm monitoring and tracking of discharged patients, a good and continuous relationship between doctors and patients was established through the WeChat platform, which should be more direct, rapid, dynamic, and friendly.

There are still some disadvantages in the network-based health information model. For example, the quality of the network was sometimes uncertain, which might cause doctors to misread or misunderstand the information. In addition, doctors cannot observe patients face-to-face, leading to a lack of physical examination and verbal communication^[25]^. Therefore, we need to explain that the WeChat platform, as a new internet tool, will become a useful assistant for clinical follow-up in China rather than a replacement for outpatient follow-up. We can obtain continuous information through dynamic monitoring via internet devices, and such information is valuable for clinical follow-up. Additionally, in response to any vague or suspicious information, patients can be recalled to the hospital for clinical or medical inspections to avoid a misdiagnosis or a missed diagnosis.

### Limitations

There are some limitations in this study. First, our research participants were children, and they did not have enough understanding of the disease and the doctors’ orders, so they needed the help of their family members for clinical follow-up or communication, which might have caused deviation and uncertainty in the results. Second, the sample size of the study was limited, and the follow-up time was short. Future research will need to confirm these conclusions in a larger cohort with longer follow-up periods and further investigate whether the WeChat follow-up model will lead to improved clinical results and whether it can reduce the burden of doctors.

## CONCLUSION

A midterm clinical follow-up for patients who underwent transthoracic device closure for a VSD through the WeChat platform can effectively reduce the time and financial burden among patients’ families and improve both medication adherence and satisfaction. This method is worthy of clinical promotion.

**Table:** 

Authors' roles & responsibilities	
W-PX	Substantial contributions to the design of the work; and the analysis of data for the work; drafting the work; final approval of the version to be published
J-FL	Substantial contributions to the design of the work; and the analysis of data for the work; drafting the work; final approval of the version to be published
Y-QL	Substantial contributions to the acquisition of data for the work; final approval of the version to be published
Z-CW	Substantial contributions to the acquisition of data for the work; final approval of the version to be published
QC	Substantial contributions to the acquisition of data for the work; final approval of the version to be published
HC	Substantial contributions to the design of the work; and the analysis of data for the work; drafting the work; final approval of the version to be published

## References

[1] Hoffman JI (1995). Incidence of congenital heart disease: I. postnatal incidence. Pediatr Cardiol.

[2] Holzer RJ, Sallehuddin A, Hijazi ZM (2016). Surgical strategies and novel alternatives for the closure of ventricular septal defects. Expert Rev Cardiovasc Ther.

[3] Yi K, You T, Ding ZH, Hou XD, Liu XG, Wang XK (2018). Comparison of transcatheter closure, mini-invasive closure, and open-heart surgical repair for treatment of perimembranous ventricular septal defects in children: a PRISMA-compliant network meta-analysis of randomized and observational studies. Medicine (Baltimore).

[4] Dai XF, Chen Q, Zhang GC, Chen LW (2020). A comparative study of minimal lower-sternal incision device closure, minimal right thoracic incision device closure, and midsternal open repair of isolated perimembranous VSD, a retrospective cohort study. Int J Cardiol.

[5] Liang W, Zhou S, Fan T, Song S, Li B, Dong H (2019). Midterm results of transaxillary occluder device closure of perimembranous ventricular septal defect guided solely by transesophageal echocardiography. Heart Surg Forum.

[6] Ergün S, Genç SB, Yildiz O, Öztürk E, Kafali HC, Ayyildiz P (2019). Risk factors for major adverse events after surgical closure of ventricular septal defect in patients less than 1 year of age: a single-center retrospective. Braz J Cardiovasc Surg.

[7] Garg P, Bishnoi AK, Lakhia K, Surti J, Siddiqui S, Solanki P (2017). Transverse sternal split: a safe mini-invasive approach for perventricular device closure of ventricular septal defect. Braz J Cardiovasc Surg.

[8] Li H, Shi Y, Zhang S, Ren Y, Rong X, Wang Z (2019). Short- and medium-term follow-up of transcatheter closure of perimembranous ventricular septal defects. BMC Cardiovasc Disord.

[9] Wiegand S, Marggraf J, Wilhelm T, Eivazi B, Werner JA (2014). Internet-mediated physician-patient interaction focusing on extracranial hemangiomas and vascular malformations. Head Neck.

[10] Zhang X, Xiao H, Chen Y (2019). Evaluation of a WeChat-based life review programme for cancer patients: a quasi-experimental study. J Adv Nurs.

[11] Feng S, Liang Z, Zhang R, Liao W, Chen Y, Fan Y (2017). Effects of mobile phone WeChat services improve adherence to corticosteroid nasal spray treatment for chronic rhinosinusitis after functional endoscopic sinus surgery: a 3-month follow-up study. Eur Arch Otorhinolaryngol.

[12] Li W, Han LQ, Guo YJ, Sun J (2016). Using WeChat official accounts to improve malaria health literacy among Chinese expatriates in Niger: an intervention study. Malar J.

[13] Lyu KX, Zhao J, Wang B, Xiong GX, Yang WQ, Liu QH (2016). Smartphone application WeChat for clinical follow-up of discharged patients with head and neck tumors: a randomized controlled trial. Chin Med J (Engl).

[14] Kim JH, Lee WY, Hong YP, Ryu WS, Lee KJ, Lee WS (2014). Psychometric properties of a short self-reported measure of medication adherence among patients with hypertension treated in a busy clinical setting in Korea. J Epidemiol.

[15] Tefera YG, Gebresillassie BM, Emiru YK, Yilma R, Hafiz F, Akalu H (2020). Diabetic health literacy and its association with glycemic control among adult patients with type 2 diabetes mellitus attending the outpatient clinic of a university hospital in Ethiopia. PLoS One.

[16] Shalansky SJ, Levy AR, Ignaszewski AP (2004). Self-reported morisky score for identifying nonadherence with cardiovascular medications. Ann Pharmacother.

[17] Murray DW, Britton AR, Bulstrode CJ (1997). Loss to follow-up matters. J Bone Joint Surg Br.

[18] Thayaparan AJ, Mahdi E (2013). The patient satisfaction questionnaire short form (PSQ-18) as an adaptable, reliable, and validated tool for use in various settings. Med Educ Online.

[19] Hafiji J, Salmon P, Hussain W (2012). Patient satisfaction with post-operative telephone calls after Mohs micrographic surgery: a New Zealand and U.K. experience. Br J Dermatol.

[20] Montag C, Becker B, Gan C (2018). The multipurpose application WeChat: a review on recent research. Front Psychol.

[21] Zhang X, Wen D, Liang J, Lei J (2017). How the public uses social media wechat to obtain health information in china: a survey study. BMC Med Inform Decis Mak.

[22] Sun ZJ, Zhu L, Liang M, Xu T, Lang JH (2016). The usability of a WeChat-based electronic questionnaire for collecting participant-reported data in female pelvic floor disorders: a comparison with the traditional paper-administered format. Menopause.

[23] Wang J, Tong Y, Jiang Y, Zhu H, Gao H, Wei R (2018). The effectiveness of extended care based on internet and home care platform for orthopaedics after hip replacement surgery in China. J Clin Nurs.

[24] Wald HS, Dube CE, Anthony DC (2007). Untangling the web--the impact of internet use on health care and the physician-patient relationship. Patient Educ Couns.

[25] Patel A, Tomar NS, Bharani A (2017). Utility of physical examination and comparison to echocardiography for cardiac diagnosis. Indian Heart J.

